# 1-(3-Chloro­phen­yl)-5-(2,4-di­hydroxy­benzo­yl)pyridin-2(1*H*)-one

**DOI:** 10.1107/S1600536813009689

**Published:** 2013-04-13

**Authors:** Fang Ren, Guifeng Li, Quanying Zhang, Jinhua Yao, Xuli Zhang

**Affiliations:** a The First Affiliated Hospital of Xinxiang Medical University, Weihui 453100, Henan Province, People’s Republic of China

## Abstract

The chloro­phenyl group of the title compound, C_18_H_12_ClNO_4_, is disordered over two orientations with occupancies of 0.331 (8) and 0.669 (8). An intra­molecular hydrogen bond is formed between a hy­droxy group and the acyclic carbonyl group. In the crystal, molecules are linked into chains along [110] by O—H⋯O and C—H⋯O hydrogen bonds, forming a ladder motif.

## Related literature

For similar structures, see: Ravinder *et al.* (2012 ▶); Sengupta *et al.* (2012 ▶). For the synthesis, see: Chen *et al.* (2011 ▶); Kim & Hong (2011 ▶). For the biological activity of similar structures, see: Kim *et al.* (2010 ▶).
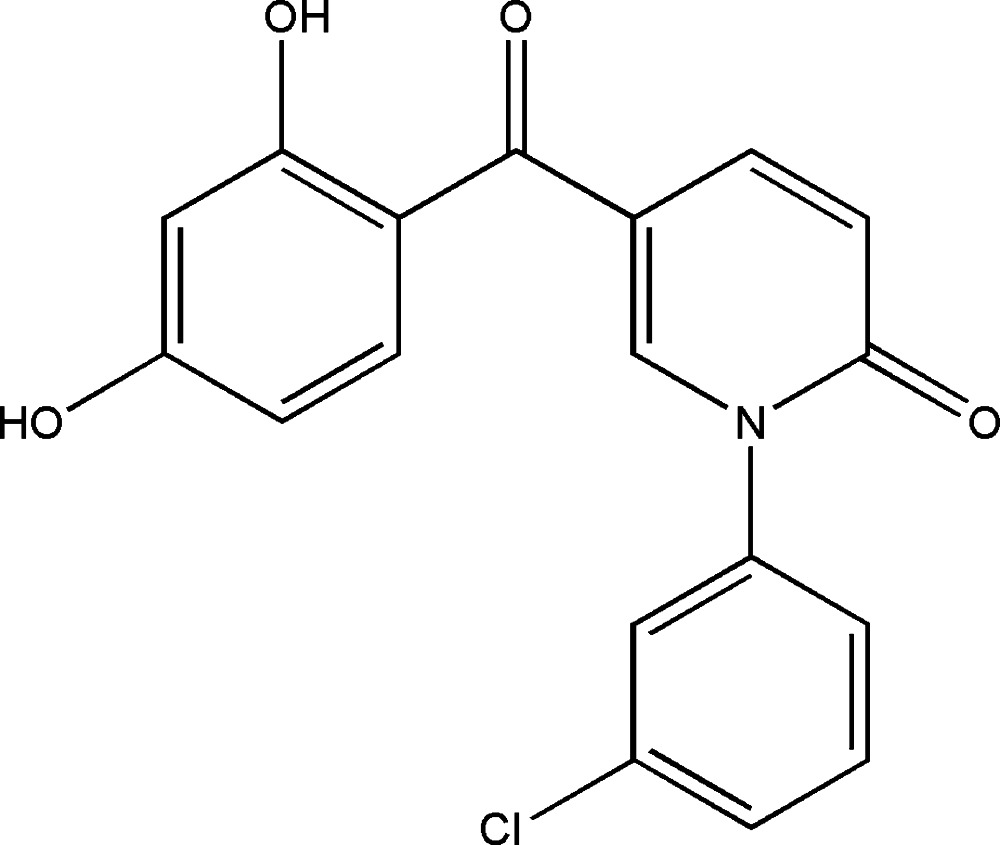


## Experimental

### 

#### Crystal data

C_18_H_12_ClNO_4_
*M* *_r_* = 341.74
Triclinic, 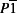

*a* = 6.689 (3) Å
*b* = 9.009 (4) Å
*c* = 13.257 (6) Å
α = 87.193 (5)°
β = 87.719 (5)°
γ = 82.674 (5)°
*V* = 791.0 (6) Å^3^
*Z* = 2
Mo *K*α radiation
μ = 0.26 mm^−1^
*T* = 293 K
0.15 × 0.12 × 0.05 mm

#### Data collection

Bruker SMART CCD diffractometer
Absorption correction: multi-scan (*SADABS*; Bruker, 2002 ▶) *T*_min_ = 0.962, *T*_max_ = 0.987
3286 measured reflections
2726 independent reflections
1929 reflections with *I* > 2σ(*I*)
*R*_int_ = 0.049

#### Refinement

*R*[*F* ^2^ > 2σ(*F* ^2^)] = 0.078
*wR*(*F* ^2^) = 0.233
*S* = 1.00
2726 reflections
236 parameters
48 restraints
H atoms treated by a mixture of independent and constrained refinement
Δρ_max_ = 0.36 e Å^−3^
Δρ_min_ = −0.43 e Å^−3^

### 

Data collection: *SMART* (Bruker, 2002 ▶); cell refinement: *SAINT* (Bruker, 2002 ▶); data reduction: *SAINT*; program(s) used to solve structure: *SHELXS97* (Sheldrick, 2008 ▶); program(s) used to refine structure: *SHELXL97* (Sheldrick, 2008 ▶); molecular graphics: *SHELXTL* (Sheldrick, 2008 ▶); software used to prepare material for publication: *SHELXTL*.

## Supplementary Material

Crystal structure: contains datablock(s) I, global. DOI: 10.1107/S1600536813009689/fy2085sup1.cif

Supplementary material file. DOI: 10.1107/S1600536813009689/fy2085Isup2.cdx

Structure factors: contains datablock(s) I. DOI: 10.1107/S1600536813009689/fy2085Isup3.hkl

Supplementary material file. DOI: 10.1107/S1600536813009689/fy2085Isup4.cml

Additional supplementary materials:  crystallographic information; 3D view; checkCIF report

## Figures and Tables

**Table 1:** Hydrogen-bond geometry (Å, °)

*D*—H⋯*A*	*D*—H	H⋯*A*	*D*⋯*A*	*D*—H⋯*A*
O2—H2⋯O3	0.91 (4)	1.75 (4)	2.587 (3)	152 (3)
O1—H1⋯O4^i^	0.87 (5)	1.79 (5)	2.655 (3)	175 (4)
C3—H3⋯O4^i^	0.93	2.50	3.165 (3)	129
C15—H15⋯O1^ii^	0.93	2.71	3.37 (2)	129

Symmetry codes: (i) 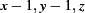; (ii) 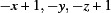.

**Fig. 1.**
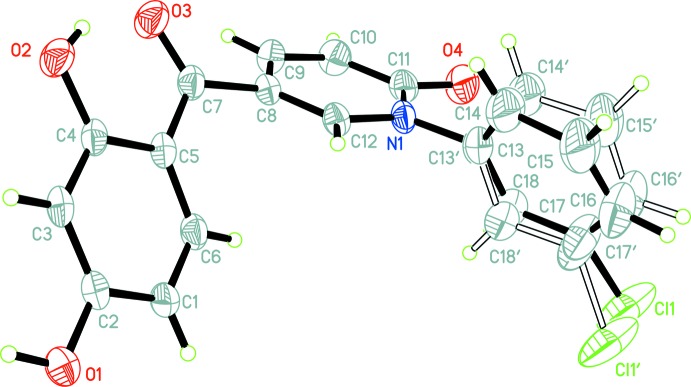
Molecular structure with the numbering scheme adopted and ellipsoids drawn at the 30% probability level.

**Fig. 2.**
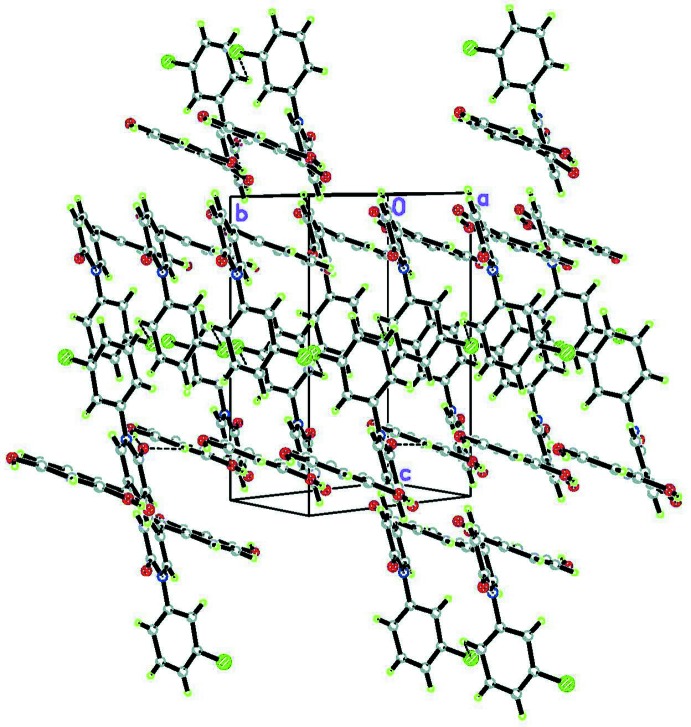
A view of the unit-cell contents of title compound. Hydrogen bonds are shown as dashed lines.

## supplementary crystallographic information

### Comment

The title compound was prepared during our ongoing research on anticancer compounds. It is one of a limited number of reported crystal structures of 2-pyridone derivatives. The structure was confirmed by X-ray crystallography as shown in Fig. 1. H-bonding interactions play a decisive role in the crystal packing arrangement (Fig. 2). Intermolecular C3—H3···O4 and O1—H1···O4 contacts form a supramolecular chain parallel with [110]. Pairs of these chains are linked via C15—H15···O1 interactions to form a ladder motif.

### Experimental

A solution of 7-methoxy-chromen-4-one (0.015 mol) in methanol (150 ml) was added dropwise to a solution of piperidine (0.030 mol) and rufluxed for 3 h at 0°C to get a crude product. This was added dropwise to a solution of dichloromethane (150 ml) and I_2_ for an overnight reaction to give 3-iodo-7-methoxy-chromen-4-one with a 70% yield. Another 100 ml round-bottom flask fitted with mechanical stirrer was loaded with a mixture of 75 ml DMF and 5 ml water, then 3-iodo-7-methyloxy-4 H-chromone (3.0 g, 0.010 mol), methyl acrylate (1.2 g, 0.015 mol), Pd(Ph_3_P)_2_Cl_2_ (0.07 g, 0.1 mmol), CuI (0.19 g, 10 mmol), and K_2_CO_3_ (1.38 g, 0.01 mol) were added successively. The mixture was heated to 70°C and stirred for 4 h. Then the mixture was filtered. The filtrate was poured into 100 ml of ice-water and then extracted with EtOAc. The extract was washed with saturated NaCl solution, dried over anhydrous MgSO_4_, and concentrated to a volume of approximately 30 ml. The target compound 3-(7-methoxy-4-oxo-4*H*-chromen-3-yl)acrylic acid methyl ester was collected after the concentrated solution was cooled down to 4°C and maintained overnight (2.1 g, 76%). The solution of 3-(7-methoxy-4-oxo-4*H*-chromen-3-yl)acrylic acid methyl ester (1.04 g, 0.004 mol), 4-chlorophenylamine (0.41 g, 0.0044 mol), and triethylamine (3 drops) in MeOH (45 ml) was stirred under reflux for 8 h. After the mixture was cooled to room temperature and the solvent removed, the crude product was purified by chromatography over silica gel to give 1-(4-chlorophenyl)-5-(2-hydroxy-4-methoxy-benzoyl)-1*H*-pyridin-2-one with a yield of 72%. Then a solution of NaOH (0.004 mol) in water (5 ml) was added dropwise to a solution of 1-(4-chlorophenyl)-5-(2-hydroxy-4-methoxy-benzoyl)-1*H*-pyridin-2-one (0.003 mol) in ethanol (50 ml). Reaction at room temperature for 10 min gave the crude compound, which was recrystallized to obtain the title compound, 1-(4-chlorophenyl)-5-(2,4-dihydroxybenzoyl)-1*H*-pyridin-2-one. The recrystallized product was dissolved in EtOAc (1.5 ml) in an ampoule and PE (1.5 ml) was added dropwise. The ampoule was placed in refrigerator and single crystals were obtained by slow evaporation of the solvent (0.3 g, 35%; m.p. 391 K).

### Refinement

All hydrogen atoms bonded with carbon atoms were placed in calculated positions using a riding model, with d(C—H) = 0.93 Å and *U*_iso_(H) = 1.2 U_eq_(C). The orientations of the hydrogen atoms in the hydroxyl groups were determined using difference Fourier maps. The disordered chlorophenyl group was divided into two parts (33%:67%). A *FLAT* group restraint and seven *ISOR* instructions (for C13-18 and Cl1) were applied for the minor component of the disordered chlorophenyl group. O—H bond distances in the two hydroxyl groups were restrained by using *DFIX* to 0.96 (1) Å.

### Crystal data

**Table:**

C_18_H_12_ClNO_4_	*F*(000) = 352
*M**_r_* = 341.74	*D*_x_ = 1.435 Mg m^−^^3^
Triclinic, *P*1	Melting point: 391 K
*a* = 6.689 (3) Å	Mo *K*α radiation, λ = 0.71073 Å
*b* = 9.009 (4) Å	Cell parameters from 841 reflections
*c* = 13.257 (6) Å	θ = 2.3–26.8°
α = 87.193 (5)°	µ = 0.26 mm^−^^1^
β = 87.719 (5)°	*T* = 293 K
γ = 82.674 (5)°	Block, yellow
*V* = 791.0 (6) Å^3^	0.15 × 0.12 × 0.05 mm
*Z* = 2	

### Data collection

**Table:**

Bruker SMART CCD diffractometer	2726 independent reflections
Radiation source: fine-focus sealed tube	1929 reflections with *I* > 2σ(*I*)
Graphite monochromator	*R*_int_ = 0.049
φ and ω scans	θ_max_ = 25.0°, θ_min_ = 1.5°
Absorption correction: multi-scan (*SADABS*; Bruker, 2002)	*h* = −5→7
*T*_min_ = 0.962, *T*_max_ = 0.987	*k* = −9→10
3286 measured reflections	*l* = −14→15

### Refinement

**Table:**

Refinement on *F*^2^	Primary atom site location: structure-invariant direct methods
Least-squares matrix: full	Secondary atom site location: difference Fourier map
*R*[*F*^2^ > 2σ(*F*^2^)] = 0.078	Hydrogen site location: inferred from neighbouring sites
*wR*(*F*^2^) = 0.233	H atoms treated by a mixture of independent and constrained refinement
*S* = 1.00	*w* = 1/[σ^2^(*F*_o_^2^) + (0.1737*P*)^2^] where *P* = (*F*_o_^2^ + 2*F*_c_^2^)/3
2726 reflections	(Δ/σ)_max_ < 0.001
236 parameters	Δρ_max_ = 0.36 e Å^−^^3^
48 restraints	Δρ_min_ = −0.43 e Å^−^^3^

### Special details

**Table:**

Geometry. All e.s.d.'s (except the e.s.d. in the dihedral angle between two l.s. planes) are estimated using the full covariance matrix. The cell e.s.d.'s are taken into account individually in the estimation of e.s.d.'s in distances, angles and torsion angles; correlations between e.s.d.'s in cell parameters are only used when they are defined by crystal symmetry. An approximate (isotropic) treatment of cell e.s.d.'s is used for estimating e.s.d.'s involving l.s. planes.
Refinement. Refinement of *F*^2^ against ALL reflections. The weighted *R*-factor *wR* and goodness of fit *S* are based on *F*^2^, conventional *R*-factors *R* are based on *F*, with *F* set to zero for negative *F*^2^. The threshold expression of *F*^2^ > σ(*F*^2^) is used only for calculating *R*-factors(gt) *etc*. and is not relevant to the choice of reflections for refinement. *R*-factors based on *F*^2^ are statistically about twice as large as those based on *F*, and *R*- factors based on ALL data will be even larger.

### Fractional atomic coordinates and isotropic or equivalent isotropic displacement parameters (Å^2^)

**Table:**

	*x*	*y*	*z*	*U*_iso_*/*U*_eq_	Occ. (<1)
C1	0.5151 (4)	−0.2487 (3)	0.2138 (2)	0.0538 (8)	
H1A	0.6295	−0.3083	0.2368	0.065*	
C2	0.3295 (4)	−0.3066 (3)	0.2132 (2)	0.0495 (7)	
C3	0.1599 (4)	−0.2167 (3)	0.1802 (2)	0.0467 (7)	
H3	0.0368	−0.2545	0.1811	0.056*	
C4	0.1723 (4)	−0.0708 (3)	0.1460 (2)	0.0437 (6)	
C5	0.3569 (4)	−0.0092 (3)	0.1467 (2)	0.0445 (6)	
C6	0.5255 (4)	−0.1048 (3)	0.1803 (2)	0.0504 (7)	
H6	0.6495	−0.0682	0.1796	0.060*	
C7	0.3660 (4)	0.1438 (3)	0.1075 (2)	0.0485 (7)	
C8	0.5392 (4)	0.2240 (3)	0.1300 (2)	0.0431 (6)	
C9	0.6104 (4)	0.3249 (3)	0.0559 (2)	0.0521 (7)	
H9	0.5547	0.3347	−0.0076	0.063*	
C10	0.7578 (4)	0.4071 (3)	0.0762 (3)	0.0608 (8)	
H10	0.8062	0.4692	0.0253	0.073*	
C11	0.8409 (4)	0.4009 (3)	0.1736 (2)	0.0554 (8)	
C12	0.6192 (4)	0.2166 (3)	0.2224 (2)	0.0460 (7)	
H12	0.5742	0.1514	0.2724	0.055*	
C13	0.835 (4)	0.298 (2)	0.348 (2)	0.0602 (8)	0.331 (8)
C14	0.679 (4)	0.326 (2)	0.4232 (19)	0.107 (3)	0.331 (8)
H14	0.5465	0.3577	0.4065	0.128*	0.331 (8)
C15	0.737 (4)	0.302 (2)	0.5263 (17)	0.137 (4)	0.331 (8)
H15	0.6406	0.3212	0.5781	0.164*	0.331 (8)
C16	0.928 (4)	0.254 (3)	0.549 (2)	0.109 (3)	0.331 (8)
H16	0.9665	0.2386	0.6154	0.131*	0.331 (8)
C17	1.065 (4)	0.227 (3)	0.471 (2)	0.0914 (18)	0.331 (8)
C18	1.027 (5)	0.242 (3)	0.364 (3)	0.069 (2)	0.331 (8)
H18	1.1232	0.2171	0.3132	0.083*	0.331 (8)
Cl1	1.3150 (14)	0.1781 (14)	0.4928 (8)	0.1598 (17)	0.331 (8)
C13'	0.8340 (19)	0.2944 (10)	0.3452 (10)	0.0602 (8)	0.669 (8)
C14'	0.7299 (15)	0.3857 (11)	0.4146 (8)	0.107 (3)	0.669 (8)
H14'	0.6124	0.4463	0.3966	0.128*	0.669 (8)
C15'	0.7991 (14)	0.3875 (11)	0.5106 (6)	0.137 (4)	0.669 (8)
H15'	0.7286	0.4493	0.5576	0.164*	0.669 (8)
C16'	0.9725 (17)	0.2980 (12)	0.5372 (8)	0.109 (3)	0.669 (8)
H16'	1.0194	0.2993	0.6022	0.131*	0.669 (8)
C17'	1.077 (2)	0.2068 (13)	0.4678 (10)	0.0914 (18)	0.669 (8)
C18'	1.007 (2)	0.2050 (12)	0.3718 (11)	0.069 (2)	0.669 (8)
H18'	1.0780	0.1432	0.3247	0.083*	0.669 (8)
Cl1'	1.2968 (6)	0.0926 (8)	0.5029 (3)	0.1598 (17)	0.669 (8)
N1	0.7644 (3)	0.3024 (2)	0.24405 (18)	0.0477 (6)	
O1	0.3277 (3)	−0.4514 (2)	0.24453 (18)	0.0642 (7)	
O2	0.0021 (3)	0.0106 (2)	0.11379 (17)	0.0591 (6)	
O3	0.2307 (3)	0.2123 (2)	0.05679 (18)	0.0686 (7)	
O4	0.9690 (3)	0.4788 (2)	0.1992 (2)	0.0816 (8)	
H2	0.044 (5)	0.096 (4)	0.086 (3)	0.077 (10)*	
H1	0.214 (7)	−0.478 (5)	0.227 (3)	0.109 (15)*	

### Atomic displacement parameters (Å^2^)

**Table:**

	*U*^11^	*U*^22^	*U*^33^	*U*^12^	*U*^13^	*U*^23^
C1	0.0483 (14)	0.0462 (14)	0.070 (2)	−0.0105 (11)	−0.0145 (13)	−0.0106 (13)
C2	0.0561 (16)	0.0472 (14)	0.0490 (18)	−0.0157 (12)	−0.0057 (12)	−0.0132 (11)
C3	0.0451 (14)	0.0543 (15)	0.0458 (17)	−0.0217 (11)	−0.0010 (11)	−0.0129 (11)
C4	0.0410 (13)	0.0532 (14)	0.0398 (16)	−0.0115 (11)	−0.0027 (10)	−0.0150 (11)
C5	0.0444 (14)	0.0506 (14)	0.0420 (16)	−0.0149 (11)	−0.0030 (10)	−0.0123 (11)
C6	0.0419 (13)	0.0519 (15)	0.061 (2)	−0.0145 (11)	−0.0059 (12)	−0.0161 (12)
C7	0.0430 (14)	0.0538 (15)	0.0516 (18)	−0.0132 (11)	−0.0041 (12)	−0.0112 (12)
C8	0.0462 (13)	0.0413 (12)	0.0442 (17)	−0.0117 (10)	−0.0031 (11)	−0.0079 (10)
C9	0.0550 (15)	0.0587 (15)	0.0453 (18)	−0.0167 (12)	−0.0046 (12)	−0.0019 (12)
C10	0.0614 (17)	0.0601 (17)	0.064 (2)	−0.0251 (14)	−0.0006 (14)	0.0066 (14)
C11	0.0520 (15)	0.0448 (14)	0.073 (2)	−0.0189 (12)	−0.0066 (13)	−0.0067 (13)
C12	0.0526 (14)	0.0428 (13)	0.0454 (18)	−0.0160 (11)	−0.0004 (12)	−0.0056 (11)
C13	0.0676 (18)	0.0643 (18)	0.052 (2)	−0.0113 (14)	−0.0134 (14)	−0.0153 (14)
C14	0.121 (7)	0.130 (8)	0.061 (4)	0.033 (6)	−0.024 (4)	−0.036 (5)
C15	0.172 (8)	0.167 (10)	0.062 (5)	0.032 (8)	−0.024 (5)	−0.051 (6)
C16	0.118 (7)	0.148 (8)	0.065 (4)	−0.024 (5)	−0.029 (4)	−0.012 (5)
C17	0.079 (3)	0.124 (4)	0.072 (3)	−0.009 (3)	−0.026 (2)	0.000 (3)
C18	0.066 (3)	0.077 (7)	0.065 (3)	−0.012 (4)	−0.009 (2)	−0.004 (4)
Cl1	0.1116 (14)	0.220 (5)	0.1318 (18)	0.034 (3)	−0.0528 (12)	0.048 (3)
C13'	0.0676 (18)	0.0643 (18)	0.052 (2)	−0.0113 (14)	−0.0134 (14)	−0.0153 (14)
C14'	0.121 (7)	0.130 (8)	0.061 (4)	0.033 (6)	−0.024 (4)	−0.036 (5)
C15'	0.172 (8)	0.167 (10)	0.062 (5)	0.032 (8)	−0.024 (5)	−0.051 (6)
C16'	0.118 (7)	0.148 (8)	0.065 (4)	−0.024 (5)	−0.029 (4)	−0.012 (5)
C17'	0.079 (3)	0.124 (4)	0.072 (3)	−0.009 (3)	−0.026 (2)	0.000 (3)
C18'	0.066 (3)	0.077 (7)	0.065 (3)	−0.012 (4)	−0.009 (2)	−0.004 (4)
Cl1'	0.1116 (14)	0.220 (5)	0.1318 (18)	0.034 (3)	−0.0528 (12)	0.048 (3)
N1	0.0537 (12)	0.0454 (11)	0.0477 (15)	−0.0153 (9)	−0.0103 (10)	−0.0097 (10)
O1	0.0658 (13)	0.0476 (11)	0.0836 (17)	−0.0193 (9)	−0.0186 (11)	−0.0013 (10)
O2	0.0421 (10)	0.0625 (12)	0.0751 (16)	−0.0144 (9)	−0.0131 (9)	0.0011 (10)
O3	0.0598 (13)	0.0624 (12)	0.0871 (18)	−0.0197 (10)	−0.0250 (11)	0.0100 (11)
O4	0.0729 (14)	0.0669 (14)	0.114 (2)	−0.0362 (11)	−0.0279 (13)	−0.0020 (13)

### Geometric parameters (Å, º)

**Table:**

C1—C6	1.359 (4)	C13—C14	1.42 (3)
C1—C2	1.407 (4)	C13—N1	1.47 (3)
C1—H1A	0.9300	C14—C15	1.43 (3)
C2—O1	1.350 (3)	C14—H14	0.9300
C2—C3	1.380 (4)	C15—C16	1.34 (3)
C3—C4	1.381 (4)	C15—H15	0.9300
C3—H3	0.9300	C16—C17	1.36 (3)
C4—O2	1.346 (3)	C16—H16	0.9300
C4—C5	1.418 (3)	C17—C18	1.45 (3)
C5—C6	1.402 (4)	C17—Cl1	1.71 (3)
C5—C7	1.457 (4)	C18—H18	0.9300
C6—H6	0.9300	C13'—C18'	1.373 (8)
C7—O3	1.232 (3)	C13'—C14'	1.373 (8)
C7—C8	1.489 (3)	C13'—N1	1.432 (13)
C8—C12	1.352 (4)	C14'—C15'	1.373 (8)
C8—C9	1.415 (4)	C14'—H14'	0.9300
C9—C10	1.349 (4)	C15'—C16'	1.373 (8)
C9—H9	0.9300	C15'—H15'	0.9300
C10—C11	1.422 (4)	C16'—C17'	1.373 (8)
C10—H10	0.9300	C16'—H16'	0.9300
C11—O4	1.242 (3)	C17'—C18'	1.373 (8)
C11—N1	1.385 (4)	C17'—Cl1'	1.750 (14)
C12—N1	1.363 (3)	C18'—H18'	0.9300
C12—H12	0.9300	O1—H1	0.87 (5)
C13—C18	1.34 (2)	O2—H2	0.91 (4)
			
C6—C1—C2	119.3 (3)	C13—C14—H14	121.6
C6—C1—H1A	120.3	C15—C14—H14	121.6
C2—C1—H1A	120.3	C16—C15—C14	120.6 (18)
O1—C2—C3	122.9 (2)	C16—C15—H15	119.7
O1—C2—C1	117.2 (3)	C14—C15—H15	119.7
C3—C2—C1	119.9 (2)	C15—C16—C17	118.1 (16)
C2—C3—C4	120.3 (2)	C15—C16—H16	120.9
C2—C3—H3	119.8	C17—C16—H16	120.9
C4—C3—H3	119.8	C16—C17—C18	127.1 (13)
O2—C4—C3	117.5 (2)	C16—C17—Cl1	121.1 (18)
O2—C4—C5	121.6 (2)	C18—C17—Cl1	111.6 (18)
C3—C4—C5	120.9 (2)	C13—C18—C17	111.2 (12)
C6—C5—C4	116.9 (2)	C13—C18—H18	124.4
C6—C5—C7	123.5 (2)	C17—C18—H18	124.4
C4—C5—C7	119.5 (2)	C18'—C13'—C14'	120.0
C1—C6—C5	122.6 (2)	C18'—C13'—N1	121.4 (8)
C1—C6—H6	118.7	C14'—C13'—N1	118.4 (7)
C5—C6—H6	118.7	C13'—C14'—C15'	120.0
O3—C7—C5	121.9 (2)	C13'—C14'—H14'	120.0
O3—C7—C8	117.7 (2)	C15'—C14'—H14'	120.0
C5—C7—C8	120.4 (2)	C16'—C15'—C14'	120.0
C12—C8—C9	117.8 (2)	C16'—C15'—H15'	120.0
C12—C8—C7	122.5 (2)	C14'—C15'—H15'	120.0
C9—C8—C7	119.4 (3)	C17'—C16'—C15'	120.0
C10—C9—C8	120.9 (3)	C17'—C16'—H16'	120.0
C10—C9—H9	119.5	C15'—C16'—H16'	120.0
C8—C9—H9	119.5	C16'—C17'—C18'	120.0
C9—C10—C11	121.5 (3)	C16'—C17'—Cl1'	119.5 (8)
C9—C10—H10	119.3	C18'—C17'—Cl1'	120.5 (8)
C11—C10—H10	119.3	C13'—C18'—C17'	120.0
O4—C11—N1	119.4 (3)	C13'—C18'—H18'	120.0
O4—C11—C10	125.1 (3)	C17'—C18'—H18'	120.0
N1—C11—C10	115.5 (2)	C12—N1—C11	122.8 (2)
C8—C12—N1	121.5 (2)	C12—N1—C13'	118.3 (4)
C8—C12—H12	119.3	C11—N1—C13'	118.9 (4)
N1—C12—H12	119.3	C12—N1—C13	119.1 (9)
C18—C13—C14	125.9 (12)	C11—N1—C13	118.0 (9)
C18—C13—N1	118.1 (18)	C2—O1—H1	108 (3)
C14—C13—N1	114.7 (18)	C4—O2—H2	104 (2)
C13—C14—C15	116.8 (16)		
			
C6—C1—C2—O1	178.2 (3)	C15—C16—C17—Cl1	175 (2)
C6—C1—C2—C3	−1.0 (4)	C14—C13—C18—C17	−5.7 (18)
O1—C2—C3—C4	−177.9 (2)	N1—C13—C18—C17	−172.1 (17)
C1—C2—C3—C4	1.2 (4)	C16—C17—C18—C13	4 (2)
C2—C3—C4—O2	179.3 (2)	Cl1—C17—C18—C13	−172.5 (17)
C2—C3—C4—C5	−1.9 (4)	C18'—C13'—C14'—C15'	0.0
O2—C4—C5—C6	−179.0 (2)	N1—C13'—C14'—C15'	−176.2 (8)
C3—C4—C5—C6	2.2 (4)	C13'—C14'—C15'—C16'	0.0
O2—C4—C5—C7	−2.9 (4)	C14'—C15'—C16'—C17'	0.0
C3—C4—C5—C7	178.4 (2)	C15'—C16'—C17'—C18'	0.0
C2—C1—C6—C5	1.4 (5)	C15'—C16'—C17'—Cl1'	−179.7 (7)
C4—C5—C6—C1	−2.0 (4)	C14'—C13'—C18'—C17'	0.0
C7—C5—C6—C1	−178.0 (3)	N1—C13'—C18'—C17'	176.0 (8)
C6—C5—C7—O3	162.1 (3)	C16'—C17'—C18'—C13'	0.0
C4—C5—C7—O3	−13.8 (4)	Cl1'—C17'—C18'—C13'	179.7 (7)
C6—C5—C7—C8	−19.3 (4)	C8—C12—N1—C11	−0.6 (4)
C4—C5—C7—C8	164.8 (2)	C8—C12—N1—C13'	176.6 (6)
O3—C7—C8—C12	136.6 (3)	C8—C12—N1—C13	175.5 (12)
C5—C7—C8—C12	−42.0 (4)	O4—C11—N1—C12	178.0 (2)
O3—C7—C8—C9	−36.4 (4)	C10—C11—N1—C12	−0.1 (4)
C5—C7—C8—C9	145.0 (3)	O4—C11—N1—C13'	0.7 (6)
C12—C8—C9—C10	2.1 (4)	C10—C11—N1—C13'	−177.3 (6)
C7—C8—C9—C10	175.4 (2)	O4—C11—N1—C13	1.8 (12)
C8—C9—C10—C11	−2.9 (4)	C10—C11—N1—C13	−176.2 (12)
C9—C10—C11—O4	−176.1 (3)	C18'—C13'—N1—C12	97.6 (6)
C9—C10—C11—N1	1.8 (4)	C14'—C13'—N1—C12	−86.3 (6)
C9—C8—C12—N1	−0.4 (4)	C18'—C13'—N1—C11	−85.0 (6)
C7—C8—C12—N1	−173.4 (2)	C14'—C13'—N1—C11	91.0 (6)
C18—C13—C14—C15	4.8 (13)	C18—C13—N1—C12	114.9 (13)
N1—C13—C14—C15	171.6 (15)	C14—C13—N1—C12	−53.0 (14)
C13—C14—C15—C16	−1.3 (16)	C18—C13—N1—C11	−68.8 (15)
C14—C15—C16—C17	0 (3)	C14—C13—N1—C11	123.3 (10)
C15—C16—C17—C18	−1 (3)		

### Hydrogen-bond geometry (Å, º)

**Table:**

*D*—H···*A*	*D*—H	H···*A*	*D*···*A*	*D*—H···*A*
O2—H2···O3	0.91 (4)	1.75 (4)	2.587 (3)	152 (3)
O1—H1···O4^i^	0.87 (5)	1.79 (5)	2.655 (3)	175 (4)
C3—H3···O4^i^	0.93	2.50	3.165 (3)	129
C15—H15···O1^ii^	0.93	2.71	3.37 (2)	129

Symmetry codes: (i) *x*−1, *y*−1, *z*; (ii) −*x*+1, −*y*, −*z*+1.
